# A chromosome-level genome assembly for the Pacific oyster *Crassostrea gigas*

**DOI:** 10.1093/gigascience/giab020

**Published:** 2021-03-25

**Authors:** Carolina Peñaloza, Alejandro P Gutierrez, Lél Eöry, Shan Wang, Ximing Guo, Alan L Archibald, Tim P Bean, Ross D Houston

**Affiliations:** The Roslin Institute and Royal (Dick) School of Veterinary Studies, The University of Edinburgh, Easter Bush Campus, Midlothian EH25 9RG, UK; The Roslin Institute and Royal (Dick) School of Veterinary Studies, The University of Edinburgh, Easter Bush Campus, Midlothian EH25 9RG, UK; The Roslin Institute and Royal (Dick) School of Veterinary Studies, The University of Edinburgh, Easter Bush Campus, Midlothian EH25 9RG, UK; Haskin Shellfish Research Laboratory, Department of Marine and Coastal Sciences, Rutgers University, 6959 Miller Avenue, Port Norris, NJ 08349, USA; Haskin Shellfish Research Laboratory, Department of Marine and Coastal Sciences, Rutgers University, 6959 Miller Avenue, Port Norris, NJ 08349, USA; The Roslin Institute and Royal (Dick) School of Veterinary Studies, The University of Edinburgh, Easter Bush Campus, Midlothian EH25 9RG, UK; The Roslin Institute and Royal (Dick) School of Veterinary Studies, The University of Edinburgh, Easter Bush Campus, Midlothian EH25 9RG, UK; The Roslin Institute and Royal (Dick) School of Veterinary Studies, The University of Edinburgh, Easter Bush Campus, Midlothian EH25 9RG, UK

**Keywords:** Pacific oyster, genome assembly, aquaculture, DNA sequencing, Hi-C chromosome conformation capture

## Abstract

**Background:**

The Pacific oyster (*Crassostrea gigas*) is a bivalve mollusc with vital roles in coastal ecosystems and aquaculture globally. While extensive genomic tools are available for *C. gigas*, highly contiguous reference genomes are required to support both fundamental and applied research. Herein we report the creation and annotation of a chromosome-level assembly for *C. gigas*.

**Findings:**

High-coverage long- and short-read sequence data generated on Pacific Biosciences and Illumina platforms were used to generate an initial assembly, which was then scaffolded into 10 pseudo-chromosomes using both Hi-C sequencing and a high-density linkage map. The assembly has a scaffold N50 of 58.4 Mb and a contig N50 of 1.8 Mb, representing a step advance on the previously published *C. gigas* assembly. Annotation based on Pacific Biosciences Iso-Seq and Illumina RNA-Seq resulted in identification of ∼30,000 putative protein-coding genes. Annotation of putative repeat elements highlighted an enrichment of *Helitron* rolling-circle transposable elements, suggesting their potential role in shaping the evolution of the *C. gigas* genome.

**Conclusions:**

This new chromosome-level assembly will be an enabling resource for genetics and genomics studies to support fundamental insight into bivalve biology, as well as for selective breeding of *C. gigas* in aquaculture.

## Data Description

### Context

The Pacific oyster, *Crassostrea gigas* (Thunberg, 1793) (NCBI:txid29159), also referred to as *Magallana gigas* by some authors [1, 2], is a keystone ecosystem and aquaculture species [3]. Although native to the Pacific coast of northeast Asia [4], *C. gigas* has been introduced to all continents, except Antarctica, for farming purposes [5–9]. The intensive human-mediated spread of Pacific oysters was mainly catalysed by the collapse of the fishery and culture of native oyster stocks due to disease, overexploitation, or other human-induced pressures, and the need to supplement depleted stocks [10, 11]. Most of these initiatives had far-reaching effects on the global distribution of Pacific oysters since several self-sustaining populations became established in the wild [12, 13]. As a result, *C. gigas* is now one of the most highly produced aquaculture species globally, and a conspicuous invasive species in many countries [14].

The extent of genetic and genomic resources developed for Pacific oysters is unparalleled among bivalve molluscs [15] and has expanded significantly in recent years. Hence, they are often used to represent Lophotrochozoa [16, 17], an understudied sister group of Ecdysozoans showing the greatest diversity of body plans among Bilaterians [18]. These resources have also been applied to enhance aquaculture production, with early technological advances in *C. gigas* focused on developing techniques to improve production through ploidy manipulation [19, 20], which later allowed the creation of the first tetraploid and triploid oyster stocks [21]. Advances in DNA sequencing technologies led to rapid additional resource development for this species, including extensive transcriptome datasets [22–26], linkage maps using microsatellite and more recently single nucleotide polymorphism (SNP) markers [27, 28], and medium- and high-density SNP arrays [29, 30]. These tools have become valuable genomic resources to enhance genetic improvement of production traits, such as growth and disease resistance, in selective breeding programmes [31, 32]. Nevertheless, a key resource for enabling genetics and genomic research in a given species is a high-quality reference genome. Zhang et al. [33] published the first draft reference genome assembly for *C. gigas* using a fosmid-pooling strategy, short-read sequencing, and a hierarchical assembly approach. Interrogation of the reference genome data pointed to gene expansion as a likely factor explaining the adaptation of *C. gigas* to challenging marine environments, a finding that has been mirrored in a number of subsequent reference genome studies for bivalve shellfish (reviewed in [34]). Although a major achievement, and indeed one of the first genome assemblies for a molluscan species, the publicly available reference genome (GenBank accession No. GCA_000297895.2) is highly fragmented, with 26,965 contigs (N50 length = 42.3 kb) and 7,655 scaffolds (N50 length = 286.8 kb). Moreover, the previous version of this assembly (GenBank accession No. GCA_000297895.1) contains many misplaced and chimeric scaffolds as revealed by alignment with linkage maps [27, 28]. These issues likely derived from a combination of both biological factors, such as the high levels of genome heterozygosity and repeat content, and technical factors, such as the reliance on short-read sequencing available at the time [33]. Therefore, highly contiguous and accurate reference genome assemblies would represent valuable resources for enabling genetics and genomic research in this keystone species.

In the present study, an improved (chromosome-level) assembly was developed for *C. gigas* by harnessing high-coverage Pacific Biosciences (PacBio) long-read sequencing (∼70×), alongside accurate Illumina short-read data (∼50×). The assembly was then scaffolded to chromosome level using both Hi-C sequencing and a high-density SNP linkage map, and the genome was annotated on the basis of both Illumina and PacBio transcript sequencing. This improved reference genome assembly represents a step towards improving our understanding of fundamental biological and evolutionary questions, and the genetic improvement of important aquaculture production traits via genomics-enabled breeding.

## Methods

### Sample collection and sequencing

A single female individual collected in 2017 from Guernsey Sea Farms (Guernsey, UK) was used for whole-genome resequencing with the PacBio Sequel (Pacific Biosciences, Menlo Park, CA, USA) and the HiSeq X (Illumina, Inc., San Diego, CA, USA) platforms. Guernsey Sea Farms is one of the primary suppliers of spat to the UK industry and has maintained lines of oysters since the early 2000s when oysters were initially imported from British Columbia (Canada) via Seasalter (Whitstable, UK). The stock was later supplemented with genetic material from the Conwy Fisheries Laboratory (UK), which was originally sourced from Japan (Miyagi, Hiroshima, and Kumamoto) and the United States (Oregon). These stocks have all been interbred with no specific maintenance of lines. High-quality, double-stranded DNA was isolated from ethanol-preserved gill tissue using a cetyltrimethylammonium bromide (CTAB) based extraction method. The DNA extraction quality was verified by the NanoDrop A260/280 and 260/230 ratios and fluorescence-based electrophoresis on a 2200 TapeStation System (Agilent Technologies, Inc., Santa Clara, CA, USA). Using this purified DNA, 3 different types of libraries were prepared to generate the sequencing data used for the assembly of the *C. gigas* genome. The first set of libraries were generated to obtain long PacBio reads and develop an initial *de novo* assembly. Two SMRTbell® libraries (chemistry v3.0) were prepared and sequenced by Edinburgh Genomics (University of Edinburgh, UK) across 13 single-molecule real-time (SMRT) cells of a PacBio Sequel system. A total of ∼55 Gb of raw bases with an N50 length of 12,777 bp were produced (Supplementary Fig. S1). Second, a paired-end sequencing library of 350 bp insert size was prepared from the same individual and then used for (i) sequence error correction, (ii) investigation of the characteristics of the genome, and (iii) quality assessment of the draft genome assembly. This library was produced by Edinburgh Genomics using the TruSeq DNA Nano gel-free library kit (Illumina) and then sequenced on a HiSeq X platform (2 × 150 bp paired-end reads). Approximately 210 million short reads were obtained after quality filtering (average base quality >15 over 5 bp) and adapter removal with Trimmomatic v0.38 [35]. Third, a Hi-C library was generated with the purpose of scaffolding the assembly into large pseudo-chromosomes. Libraries were prepared using the Dovetail^TM^ Hi-C Library Preparation Kit, following the manufacturer's protocol (Dovetail™ Hi-C Kit Manual v.1.03). The genomic DNA used for the Hi-C library came from snap-frozen gill tissue sampled from the same individual described above. This final library was sequenced on an Illumina HiSeq X platform (2 × 150 bp reads) and resulted in 500 million read pairs.

Total RNA was extracted from 2 additional individual oysters (also from Guernsey Sea Farms, Guernsey, UK), a male and a female, from 6 distinct tissues (gill, mantle, digestive gland, heart, adductor muscle, and gonads). Full-length transcripts were isolated from the tissue samples using a combination of the TRIzol (Invitrogen) and the RNeasy Plus Mini kit (Qiagen) protocols, with the inclusion of a DNAse treatment step. RNA quality was assessed using the Nanodrop ND-1000 and the Agilent 2200 TapeStation instruments. RNA extracts were quantified using a Qubit^TM^ RNA assay kit (Thermo Fisher, Waltham, MA, USA) and then combined in equimolar quantities into a single pool for sequencing. The final RNA pool was used to obtain full-length coding DNA (cDNA) sequences using the TeloPrime Full-Length cDNA Amplification Kit v2 (Lexogen). cDNA was then sequenced across 3 SMRT cells of a PacBio Sequel platform at the Dresden-concept Genome Center (Germany). A total of 178 Gb of data comprising 1.6 million transcripts with a mean length of 1.3 kb were generated for gene annotation.

### Genome features

Owing to the differences in genome size estimates reported in the literature for *C. gigas* [15, 33], the DNA content of the Pacific oyster genome was also estimated in the present study. To this end, the genome size was determined for the sequenced female using a *k*-mer-based approach and flow cytometry. For the *k*-mer analysis, quality-filtered Illumina reads (150 bp length) were used to count the frequency of different *k*-mer sizes, ranging from 15 to 23, using Jellyfish v2.1.3 [36]. All *k*-values evaluated showed a clear bimodal distribution, with peaks occurring at a read depth of 19 and 37× (Supplementary Fig. S2). The *k*-mer frequency plots obtained are characteristic of species with highly heterozygous genomes [37]. From the *k*-mer–based analysis (*k*-mer = 21), the *C. gigas* genome size was estimated at 534 Mb. For the genome size estimation by flow cytometry, Pacific oyster nuclei were isolated and stained with propidium iodide [38]. Two species were used as internal standards for the assay, fruit fly (*Drosophila melanogaster*) and zebrafish (*Danio rerio*). According to flow cytometry, the genome size of the female oyster sequenced in the present study was estimated at 640 Mb. The *k*-mer–based analysis inferred a comparatively smaller genome than flow cytometry, which might reflect an underestimation of size in the sequence-based approach due to high heterozygosity and repeat content [39]. Hence, the flow cytometry measurement was used as the reference size to calculate the predicted sequencing yield and anticipated length for *de novo* genome assembly. The Pacific oyster genome heterozygosity was assessed with GenomeScope v2.0 (GenomeScope, RRID:SCR_017014) [40], based on the quality-filtered Illumina reads. A heterozygosity rate of 3.2% was estimated from the 21-mer–based assessment of the oyster genome (Supplementary Fig. S3). This value is higher than the 1.3% previously reported for this species [33], which may be explained by the fact that the authors used an inbred individual for genome assembly, whereas in this study, an outbred female was sequenced. Although high, the heterozygosity value is in the range with those reported for other bivalve molluscs (e.g., 2.4% in the quagga mussel [41]).

### Genome assembly

The PacBio reads were first assembled into contigs using Canu v1.8 (Canu, RRID:SCR_015880) [42] at near default parameter values (corrected error rate = 0.045 and raw error rate = 0.300). Contigs were polished with 1 round of Arrow [43] followed by an additional round of polishing with Pilon (Pilon, RRID:SCR_014731) [44], after alignment of the post–quality-filtered Illumina reads with Minimap2 v.2.2.15 (Minimap2, RRID:SCR_018550) [45]. Compared with the genome size estimate of 640 Mb, the initially assembled version of the genome was ∼2 times larger than expected, yielding 6,368 contigs, a total length of ∼1.2 Gb, and an N50 length of 0.46 Mb. These results can be explained by the high frequency of highly divergent haplotypes in the *C. gigas* genome, a feature that has also been observed in the process of creating genome assemblies for other molluscan species [46, 47]. Whilst the size of the assembled sequence could indicate that the high level of heterozygosity had allowed the resolution of the 2 haplotypes present, we sought to establish a high-quality pseudo-haploid genome as a reference. To assess the level of duplication in the initial assembly, a BUSCO (v2.0) analysis was performed (BUSCO, RRID:SCR_015008) [48]. By searching against the metazoa_odb9 database using sea hare as a reference species, 791 BUSCO genes (80.9%) were found to be duplicated. To remove potentially redundant contigs by retaining only 1 variant of a pair of divergent haplotypes, 2 independent approaches were taken. First, the short-read data were used to identify and reassign putative haplotigs with the Purge Haplotigs pipeline (-l 5, -m 38, -h 90) [49]. Second, an all-versus-all contig mapping was performed on the repeat-masked assembly with Minimap2 v.2.2.15 (Minimap2, RRID:SCR_018550) [45]. Contigs were ordered based on their length, and matching contigs that mapped ≥30% of their length and were longer than 10 kb were removed as potential haplotigs. The reference sequence and the mapping sequences were all removed before the next iteration. The lists of curated contigs obtained independently from both methods were compared and the common contigs then selected for an additional round of haplotig purging. This approach resulted in a significant reduction in the number of contigs to 1,235, which were retained for scaffolding.

### Chromosome-level assembly using Hi-C and linkage map data

**Figure 1:**
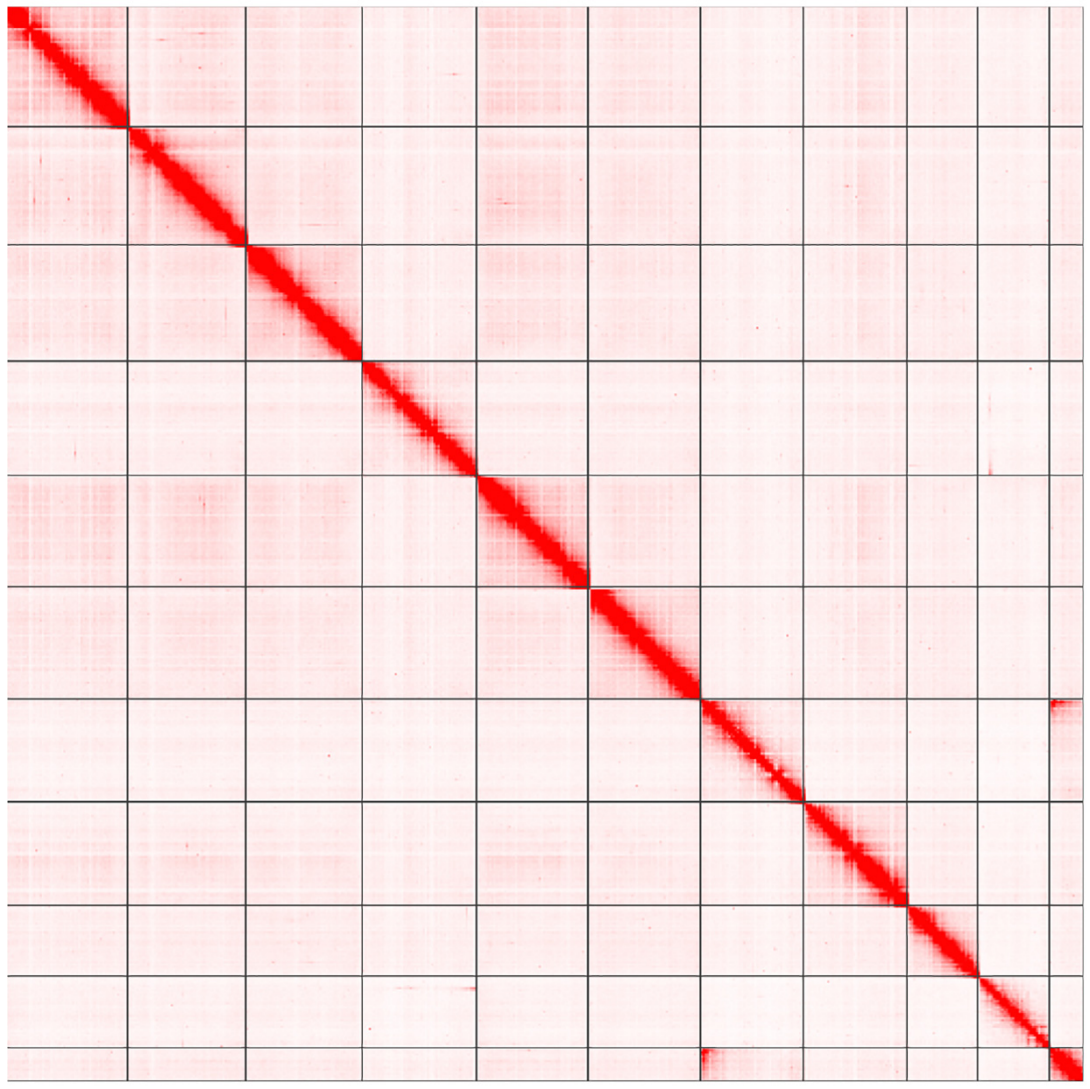
Hi-C interaction analysis depicting the 11 super-scaffolds obtained after using the HiRise™ scaffolding software. The Hi-C contact map is visualized using Juicebox v1.11.08 [57].

**Figure 2:**
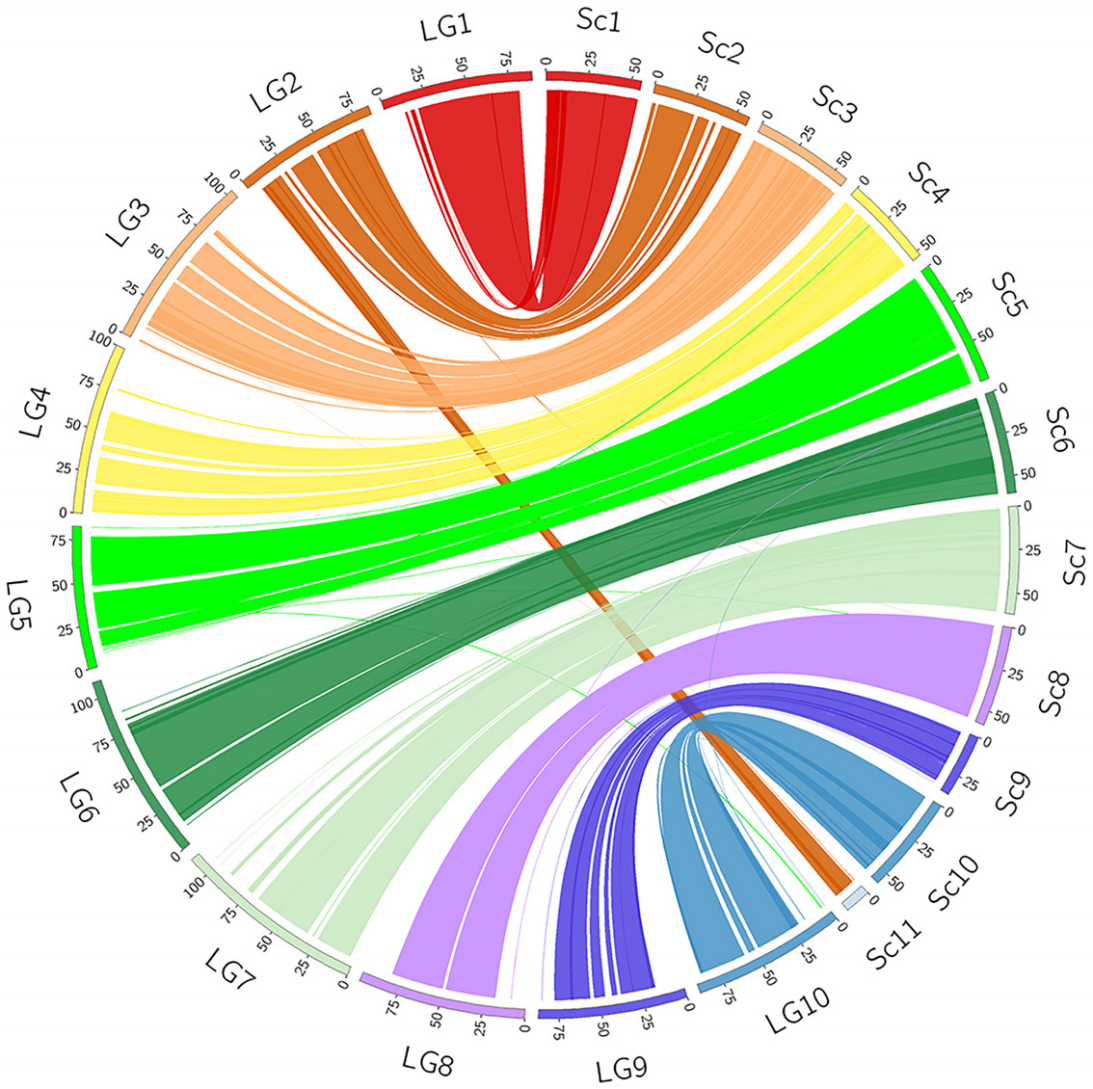
A high concordance between the chromosome-level scaffolds and a high-density linkage map allowed the anchoring of 2 scaffolds (Sc2 and Sc11) to a single linkage group 2 (LG2). Scaffold (Sc) unit lengths are in Mb. Linkage group (LG) units of distance are expressed in cM. Ticks in each linkage group or scaffold indicate lengths in 25 cM or Mb, respectively. Plot generated using Circos v0.69–8 (Circos, RRID:SCR_011798) [58].

To generate a chromosome-level assembly for *C. gigas*, Hi-C proximity ligation [50] data were used to order and orient the contigs along chromosomes. The scaffolding process was carried out by Dovetail Genomics (Santa Cruz, CA, USA) using the Dovetail^TM^ Hi-C library reads to connect and order the input set of contigs. After scaffolding with HiRise v2.1.7 [51], the assembled genome sequence initially comprised a total of ∼633 Mb, with a scaffold and contig N50 of 57.4 and 0.7 Mb, respectively. A high fraction of the assembled sequences (>92%) was contained in only 11 super-scaffolds (Fig. 1). However, Pacific oysters have 10 pairs of chromosomes [52]. A high-density linkage map [27] was used to anchor the super-scaffolds into chromosomes. SNP probes were mapped to the reference genome assembly using BWA v0.78 (BWA, RRID:SCR_010910) [53]. Of the 20,353 markers on the genetic map, 17,747 mapped to a chromosome-level scaffold with a MAPQ >16. The integration of genetic linkage information enabled the anchoring of 2 super-scaffolds onto a single linkage group (LG2), resulting in an assembly with 10 major scaffolds representing all oyster chromosomes (Fig. 2). Gaps were closed with PBJelly (PBJelly, RRID:SCR_012091) [54] and again error corrected using the short-read Illumina data using Pilon [44]. From the remaining set of unplaced scaffolds, regions of low sequence accuracy were identified on the basis of short-read coverage, following [55]. Briefly, the median read-depth per 1,000 bp (non-overlapping) windows was calculated after GC-content normalization. Scaffolds with >70% of windows showing a median coverage of 2 SD above or below the mean were removed from the analysis. All unplaced contigs and scaffolds showing significant sequence identity with the Iso-Seq data were added to the primary set.

The final Pacific oyster assembly (GenBank accession No. GCA_902806645.1) contains the 10 expected chromosomes and 226 unplaced scaffolds, with a total N50 of 58.4 and 1.8 Mb for scaffold and contig lengths, respectively (Table 1). This final assembly is 647 Mb in size, with the chromosome-level scaffolds represented in 589 Mb of sequence. In addition, the complete mitochondrial genome of *C. gigas* was assembled and is available online in the Mendeley Data repository [56]. This assembly represents a step improvement over the previous version of the *C. gigas* reference genome [33] and other oyster assemblies [47]. However, it should be noted that a separate chromosome-level reference genome assembly from the Institute of Oceanology, Chinese Academy of Sciences is available in GenBank (accession No. GCA_011032805.1). This assembly is slightly shorter at 586 Mb and has a similar scaffold N50 of 60 Mb and a higher contig N50 of 3.1 Mb. Future comparisons between these 2 high-quality assemblies will be important to evaluate their consistency and ensure uniform use of nomenclature to describe chromosomes. Furthermore, it is expected that additional high-quality reference genome assemblies will become available for this species, and the availability of multiple assemblies is advantageous for *C. gigas* as a species with high levels of intra- and inter-population genetic diversity [15]. To aid with the future coordination of this assembly with existing and future assemblies, the 10 large scaffolds described herein were aligned with the Pacific oyster karyotype using fluorescence *in situ* hydridization probes corresponding to bacterial artificial chromosome (BAC) clones (Supplementary Note A). The correspondence between the nomenclature of the linkage groups and scaffolds assembled in the present study and the nomenclature of the karyotype chromosomes is given in Supplementary Table S1. This information should enable consistency in nomenclature when describing multiple genome assemblies for this species in the future.

**Table 1:** Genome assembly statistics and annotation of *C. gigas*

Genome assembly	Value
**Genome**	
GC content (%)	33.25
Total size (bp)	647,887,097
Contigs	
No.	711
N50 length (bp)	1,813,842
Longest (bp)	11,935,632
Scaffolds	
No.	236
N50 length (bp)	58,462,999
Longest (bp)	73,550,375
**Genome annotation**	
No. transposable elements	
LTR	22,828
LINE	41,781
DNA transposons	634,611
Total	699,220
Protein-coding genes	
No.	30,724
Mean length (bp)	
Spliced transcript	2,021
Coding sequence	1,335
Exon	375
Functional annotation	
GO	18,750
KO	11,390

GO: Gene Ontology annotation; KO: KEGG Orthology annotation; LINE: long interspersed nuclear element; LTR: long terminal repeat.

### Quality assessment of reference genome

First, the *C. gigas* genome assembly was screened for contaminant DNA from a different taxon using Conterminator v1.c74b5 [59]. The search was performed against the nt NCBI database (downloaded December 2020) by ignoring unclassified sequences (NCBI:txid12908), other sequences (NCBI:txid28384), and artificial sequences (NCBI:txid81077). No evidence of contamination with foreign DNA from a different taxon was detected in the assembly. Second, to assess the completeness of the assembled genome, a BUSCO analysis was performed. From the curated list of single-copy genes, 935 (95.6%) were found in the assembly, of which 919 (94%) were single-copy and 16 (1.6%) were duplicated. Finally, to evaluate the accuracy of the reconstructed *C. gigas* genome, structural variants were called with Sniffles (Sniffles, RRID:SCR_017619) [60], after alignment of the PacBio raw reads with ngmlr v0.2.7 (Ngmlr, RRID:SCR_017620). Variants with a minimum size of 50 bp for which the ratio of high-quality reads for the assembly (reference) variant was <0.2 were considered assembly errors (Supplementary Table S2).

### Genome annotation

Genome annotation was carried out using long-read PacBio Iso-Seq data from 6 tissues and the Illumina short-read RNA-Seq data from Zhang et al. [33]. Short-read data were mapped to the reference assembly with STAR v.2.5.1b (STAR, RRID:SCR_015899) [61]. Transcript models were created by BRAKER v.2.1.5 (BRAKER, RRID:SCR_018964) [62] using only the paired-end RNA-seq datasets (see Supplementary Table S3). Multi-exon transcripts expressed in ≥2 tissues at an expression level >1 transcript per kilobase million were retained. Iso-Seq raw sub-reads were processed with SMRT Link v7.0 (SMRT-Analysis, RRID:SCR_002942) (Pacific Biosciences) to obtain circular consensus sequences (CCS) using a “–min-rq of 0.9”. The Iso-Seq CCS reads were mapped with Minimap2 v.2.16 (Minimap2, RRID:SCR_018550) [45], and the transcript models were called using the TAMA package [63] (see Supplementary Note B). Protein-coding transcripts and translation start and end positions were predicted by mapping known protein sequences from UniRef90 [64] to the oyster transcripts by Diamond v.0.9.31 (DIAMOND, RRID:SCR_016071) [65]. Those models that contained a frameshift within the coding sequence were classified as pseudo-genes.

The final annotation of the assembled *C. gigas* genome contains 35,422 genes, of which 30,724 are protein-coding, 4,000 represent non-coding RNA genes, and 698 were classified as pseudo-genes. Among the protein-coding genes, 15,646 (51%) contained putative alternative spliced transcripts, with a mean of 3.1 transcripts per gene. The gene models predicted for *C. gigas* were functionally annotated using the Blast2GO pipeline (Blast2GO, RRID:SCR_005828) [66] and KEGG orthology (KO) groups were assigned using KOBAS v2.0 (KOBAS, RRID:SCR_006350) [67]. Approximately 18,750 (61%) of the predicted protein-coding genes were assigned functional labels (Table 1). This reference genome assembly has also been annotated by the NCBI annotation team, who used the extensive short-read transcriptome data available for *C. gigas* to annotate 38,296 genes (31,371 protein-coding, 6,837 non-coding, 88 pseudo-genes) and a total of 73,946 transcripts [68].

### Repeat element annotation

Known Pacific oyster–specific repeat sequences were identified in the genome assembly using RepeatMasker v.4.0.7 (RepeatMasker, RRID:SCR_012954) [69] with a combined repeat database (Dfam_Consensus-20170127 and RepBase-20170127) [70, 71] with parameters “-s -species “Crassostrea gigas" -e ncbi”. Besides the 972 repeat families contained in the RepeatMasker library an additional 1,827 novel repeat families were identified by RepeatModeler v.1.0.11 (RepeatModeler, RRID:SCR_015027) [72]. This novel repeat library was used to identify the location of novel elements in the newly built assembly. For comparison, the same search was performed on the older version of the *C. gigas* genome assembly (GenBank assembly accession GCA_000297895.2).

**Figure 3:**
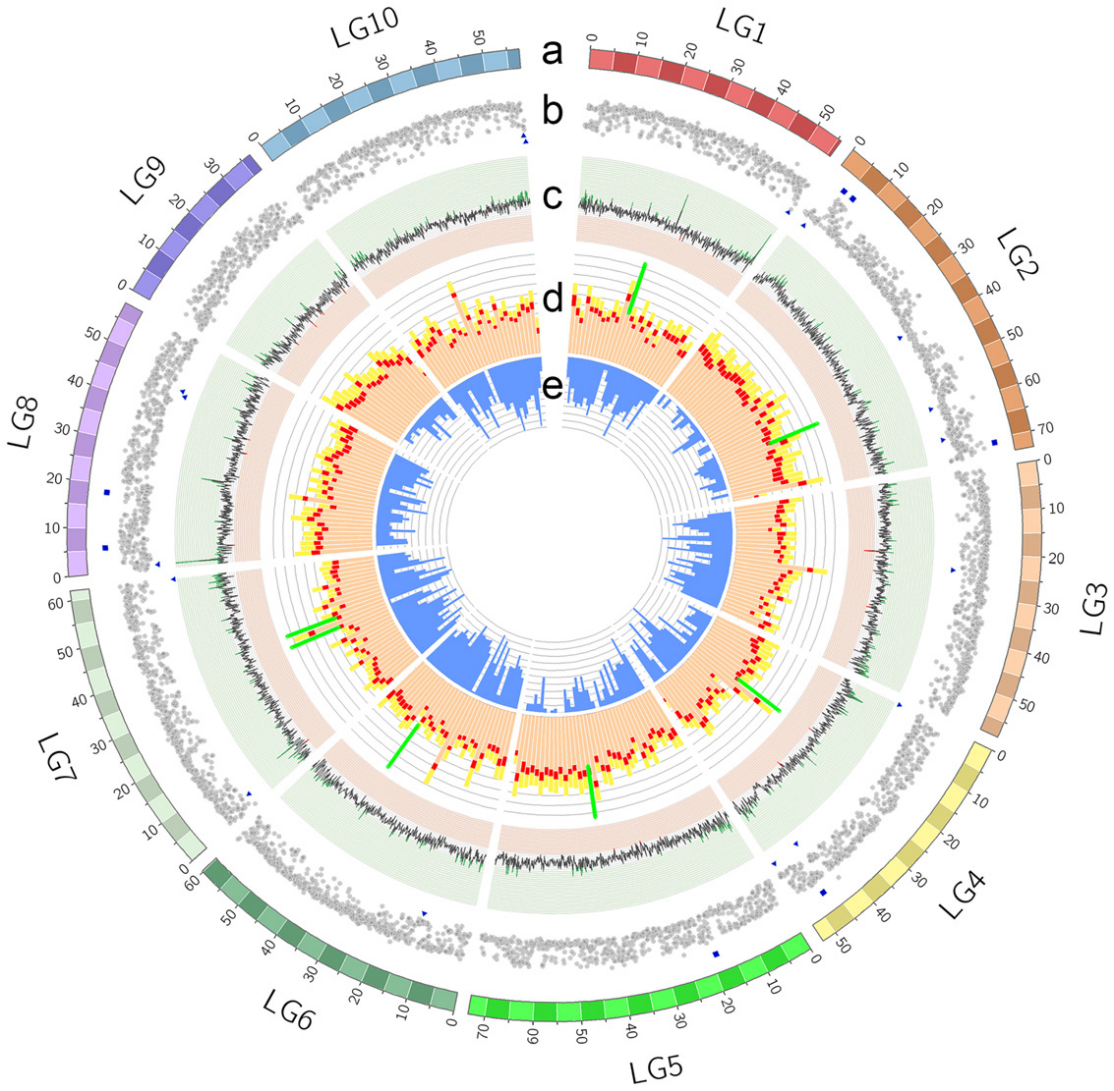
Circos plot depicting genome features across the 10 oyster chromosomes. (a) Oyster chromosomes (LG1–LG10 on an Mb scale). (b) Short-read coverage plot. Coverage within 2 SD of the mean is shown as grey circles. Abnormal sequence coverage (±2 SD from the mean) is indicated with a blue square or triangle, respectively. (c) GC content percentage (>35% in green; <31% in red). (d) Distribution of repeat elements: DNA transposons (light orange bar), retrotransposon TEs (red bar), and novel repeat elements (yellow bar). The location of centromeres is indicated with a green line. (e) Gene density (range: 50–150). For tracks (b) and (c), a window size of 0.1 Mb was used, whereas for tracks (d) and (e), the size was increased to 0.2 Mb.

Overall, a higher number of repetitive elements were identified in our assembly compared to the previous genome assembly (Supplementary Fig. S5). Repeat elements constituted 43% of the Pacific oyster genome. Repetitive sequences were distributed unevenly along the *C. gigas* chromosomes. In general, an inverse relationship between the total number of repeat elements and gene density was observed across 100-kb (non-overlapping) genomic windows in the chromosome-level scaffolds (Fig. 3d and e). If a genomic feature overlapped 2 windows, the feature was counted towards the interval with the highest length coverage. Among the different classes of repeat elements, significant negative correlations were found between gene density and (i) retrotransposons of the long terminal repeat (LTR) type (corr = −0.61; *P* = 2.2 × 10^−16^), (ii) non-LTR retrotransposons (corr = −0.28; *P* = 5.4 × 10^−7^), (iii) satellite DNA (corr = −0.29; *P* = 4.5 × 10^−7^), (iv) simple repeats (corr = −0.33; *P* = 4.7 × 10^−9^), and (v) DNA transposons (corr = −0.59; *P* = 2.2 × 10^−16^). The centromeres of 5 metacentric chromosomes were located after aligning 6 centromere-associated microsatellite markers to the assembly [73] (Supplementary Table S4). Of these 5 centromere regions, 4 co-localize with genomic windows enriched for repetitive elements (Fig. 3d). Among repetitive elements, transposable elements (TEs) were the most common and accounted for 36% of the assembled genome. Consistent with previous studies [47], the oyster genome is dominated by DNA transposons (32% of the genome assembly) (Table 1), with *Helitrons* being the most abundant superfamily (Supplementary Figs S6 and S7).

### Characterization of *Helitrons* in the Pacific oyster genome

**Figure 4:**
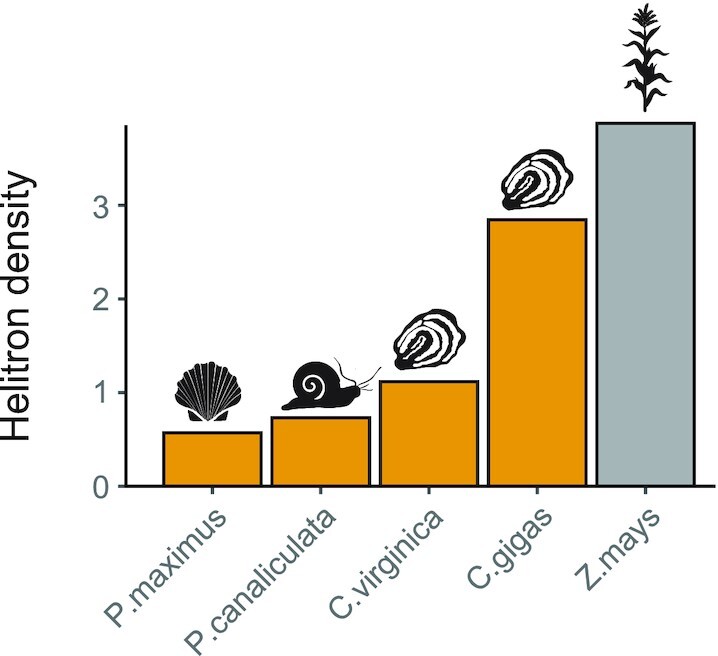
Density of *Helitrons* identified across 4 molluscan genomes (orange bars), including maize as a reference species (grey bar). The reference genome assembled for *C. gigas* was compared to the king scallop (*Pecten maximus*; GCF_902652985.1), golden apple snail (*Pomacea canaliculata*; GCF_003073045.1), and Atlantic oyster (*Crassostrea virginica*; GCF_002022765.2), with maize included as a reference species (*Zea mays*; GCF_000005005.2). *Helitron* density is expressed as the number of conserved 3′-ends over genome size (in Mb).

*Helitrons* are rolling-circle transposable elements that have the ability to capture host gene fragments [74]. In maize, *Helitrons* have significantly influenced genome evolution, leading to genome variation among lines [75] and a notable diversification of transcripts via exon shuffling of thousands of genes [76]. To refine the annotation of Pacific oyster *Helitrons*, a structure-based search [77] was performed in addition to the homology-based approach described above. The localization of these elements was heterogeneous across the Pacific oyster chromosomes, with LG5 and LG8 showing a higher density of elements (>1 SD above the average across chromosomes) (Supplementary Fig. S8). *Helitrons* in plant and animal genomes tend to accumulate in gene-poor regions [78]. However, this bias is less evident in *C. gigas*, with no significant association found between gene density and the number of *Helitrons* within a region. A comparison with other molluscan reference genome assemblies revealed that *C. gigas* had a remarkably high number of predicted *Helitron*-related sequences (Fig. 4).

The Pacific oyster *Helitron-*like sequences possess the basic expected structure observed in other taxa: TC sequence at the 5′-termini, CTAG motif on the 3′-terminus, and a 16–20 bp palindromic sequence that can form a hairpin structure upstream of the 3′-end. Likewise, they were also found to preferentially insert (86% of the cases) between the 5′-A and 3′-T nucleotides of the host AT target sites. Of the 751 intact *Helitrons* discovered through the *in silico* screening, 629 elements had a high 3′-end pairwise sequence similarity (>80% identity over 30 bp), as indicated by the clustering of sequences with vsearch v1.8.1 (-id 0.80 -iddef 1) [79], suggesting that they belong to the same family [78]. Notably, a significant fraction of these elements (257 of 751) had sub-terminal inverted repeats (subTIRs), as revealed by a screening of their paired terminal ends with the Inverted Repeats Database [87]. This structural feature is characteristic of an alternative variant of *Helitrons* called *Helentrons*, which in their non-autonomous form known as HINEs (*Helentron*-associated interspersed elements) [80] have been recently linked to the widespread dispersal of satellite DNA-like repeats in the oyster genome [81]. A search for the typical substructures reported for the oyster HINEs—e.g., subTIR, IR (complementary to the subTIR), and a microsatellite in the 5′-end—showed that a significant fraction (33%) of the elements exhibiting subTIRs also carried an IR at their 5′-end, however, only 1 had a microsatellite (see Supplementary Note C). Therefore, these elements display structural features of both *Helitrons* and *Helentrons* and may represent evolutionary intermediates, although confirming this would require further investigation.

*Helitrons* have been observed to capture gene fragments in species such as maize and the little brown bat (*Myotis lucifugus*) [82, 83]. In *C. gigas*, a BLASTX (BLASTX, RRID:SCR_001653) [84] search against the UniRef database revealed that only 17 *Helitrons* (2%) carried gene fragments; alignment lengths >50 with ≥85% identity were considered a match. The Pacific oyster *Helitron*-like sequences were relatively short (mean = 1,092 bp; SD = 558 bp) and lacked the distinctive features of autonomous elements (i.e., Rep protein motif and DNA helicase domain). Non-autonomous *Helitrons* require the enzymatic machinery of their autonomous counterparts in order to amplify. Owing to the fact that this study did not detect evidence for the presence of autonomous mobile sequences in the Pacific oyster genome, these abundant *Helitron* elements are likely to be inactive, suggesting that they are remnants of high levels of past activity in the evolutionary history of *C. gigas*.

## Conclusion

The new chromosome-level *C. gigas* genome assembly presented herein has a scaffold N50 of 58.4 Mb and a contig N50 of 1.8 Mb, representing a step advance on the previously published assembly, and will complement other high-quality assemblies available or becoming available in the near future. Approximately 30,000 putative protein-coding genes were identified with an average of 3.1 transcripts per gene. DNA transposons dominated the repeat elements detected in the assembly, with *Helitrons* being found at a substantially higher level than in other molluscan species, suggesting their potential role in shaping the evolution of the *C. gigas* genome. The availability of a chromosome-level genome assembly is expected to support applied and fundamental research in this keystone ecological and aquaculture species.

## Data Availability

The raw sequencing data underlying this article have been submitted to the European Nucleotide Archive (ENA) and can be accessed with accession No. PRJEB35351. The genomic short-read data can be accessed with accessions Nos. ERX3728455, ERX3728453, ERX3728482, ERX3728546, ERX3728630, and ERX3728636; the raw reads of the Hi-C library can be accessed with accession No. ERX3722775. PacBio Iso-Seq reads of pooled samples can be accessed with accession Nos. ERX3721883, ERX3722678, and ERX3722679. Raw PacBio reads from the nuclear DNA can be accessed with accessions Nos. ERX3761471, ERX3761586, ERX3761587, ERX3761621, ERX3761714, ERX3761715, ERX3761720, ERX3762151, ERX3762342, ERX3762370, ERX3762371, ERX3762372, and ERX3762598. The complete mitochondrial genome is hosted in Mendeley Data [85]. The Pacific oyster genome assembly is available at GenBank and can be accessed with accession No. GCA_902806645.1. Other supporting data, including the annotation of the Pacific oyster genome and BUSCO tables, are provided via the GigaScience database, GigaDB [86].

## Additional Files

Supplementary Figure S1. Read length distribution of raw PacBio reads.

Supplementary Figure S2. Distribution of different k-mer depths.

Supplementary Figure S3. GenomeScope results plots.

Supplementary Figure S4. A karyotype for *C. gigas*.

Supplementary Figure S5. Major categories of repeat elements in the genome assembly .

Supplementary Figure S6. No. of different DNA transposons identified in the genome assembly.

Supplementary Figure S7. No. of different retrotransposon TEs identified in the genome assembly.

Supplementary Figure S8. Location of putative *Helitrons*.

Supplementary Table S1. Correspondence between *C. gigas* linkage groups and chromosomes.

Supplementary Table S2. Validation of the *C. gigas* genome assembly based on long read alignment.

Supplementary Table S3. Paired-end RNA-seq read information.

Supplementary Table S4. Details of centromere-associated microsatellites.

Supplementary Note A. Integration of the genome assembly sequence with a cytogenetic map.

Supplementary Note B. Oyster genome annotation.

Supplementary Note C. Identification of *HINE* substructures in *Helitron*-like elements.

## Abbreviations

BAC: bacterial artificial chromosome; BLAST: Basic Local Alignment Search Tool; bp: base pairs; BUSCO: Benchmarking Universal Single-Copy Orthologs; BWA: Burrows-Wheeler Aligner; CCS: circular consensus sequence; cDNA: coding DNA; cM: centimorgan; Gb: gigabase pairs; GC: guanine-cytosine; kb: kilobase pairs; KEGG: Kyoto Encyclopedia of Genes and Genomes; KO: KEGG orthology; LTR: long terminal repeat; MAPQ: mapping quality; Mb: megabase pairs; N50: median size; NCBI: National Center for Biotechnology Information; PacBio: Pacific Biosciences; RNA-Seq: RNA sequencing; SNP: single nucleotide polymorphism; SMRT: single-molecule real-time; sub-TIR: sub-terminal inverted repeat.

## Competing Interests

The authors declare that they have no competing interests.

## Funding

This work was supported by funding from the Natural Environment Research Council (NE/P010695/1) and Biotechnology and Biological Sciences Research Council (BB/S004343/1, BB/P013759/1, BB/P013740/1, BB/P013732/1). The cytogenetic mapping of BACs was supported by a grant from the U.S. Department of Agriculture (2009–35 205-05 052).

## Authors' Contributions

R.D.H, C.P., and A.P.G. conceived the project; T.P.B. collected the samples; C.P. and A.P.G. performed laboratory experiments; L.E., A.P.G., C.P., and A.L.A. constructed and analysed the assembly; A.P.G. generated the linkage map; X.G. and S.W. generated the cytogenetic map; and C.P., L.E., and R.D.H. wrote the manuscript with input from all authors.

## Supplementary Material

giab020_GIGA-D-20-00306_Original_Submission

giab020_GIGA-D-20-00306_Revision_1

giab020_Response_to_Reviewer_Comments_Original_Submission

giab020_Reviewer_1_Report_Original_Submission Takeshi Takeuchi, Ph.D -- 11/9/2020 Reviewed

giab020_Reviewer_1_Report_Revision_1 Takeshi Takeuchi, Ph.D -- 2/15/2021 Reviewed

giab020_Reviewer_2_Report_Original_Submission Daniel Powell -- 11/15/2020 Reviewed

giab020_Supplemental_File
