# Supplementary material for: A chromosome-level genome assembly for the Pacific oyster Crassostrea gigas
Source: Gigascience. 2021 Mar 25;10(3):giab020. doi: 10.1093/gigascience/giab020 (PMC7992393; doi:10.1093/gigascience/giab020)
Supplement: giab020_Response_to_Reviewer_Comments_Original_Submission [file giab020_response_to_reviewer_comments_original_submission.pdf]

Author's Response To Reviewer Comments

Close

Reviewer reports:
Reviewer #1: Peñaloza et al. sequenced the genome of the Pacific oyster Crasostrea gigas using PacBio long-read sequencing technology and established a chromosome-level assembly by applying Hi-C scaffolding and a high density SNP linkage map. The genome assembly is significantly contiguous compare to previously-published draft assembly of the same species. This new reference genome is a desirable resource, therefore worth to be published in the journal GigaScience after resolving some concerns below.

1) Genome size (P.7)
I don't think it is reasonable to take the midpoint between the results of the k-mer analysis and the flow cytometry measurement. K-mer analysis can considerably underestimate genome size if the genome is rich in repeat sequences, because repeats increase mean coverage of k-mers. Thus, the genome size of 640Mb, measured experimentally, should be more reliable.

Response: We agree with the reviewers comment. The sentence "Due to the different genome size estimates obtained by the two methods, the midpoint – i.e. ~590 Mb - was used to calculate the predicted sequencing yield and anticipated length for de novo genome assembly" was removed from the manuscript.
The following sentence was added to the "Methods" section, "Genome features" sub-section in replacement:
"A comparatively lower genome size was inferred from the k-mer based analysis compared to flow cytometry, which could reflect an underestimation of size by the sequence-based approach due to high heterozygosity and repeat content (Pflug, Holmes et al. 2020). Hence, the flow cytometry measurement was used as the reference size to calculate the predicted sequencing yield and anticipated length for de novo genome assembly."

2) Assessment of DNA contamination (P.11)
As the genomic DNA was extracted from the gill tissue, where considerable amount of microorganisms may be trapped by the filter-feeding bivalve, contaminant DNA sequences should be carefully removed from the raw reads or the assembly if present.
In order to check contamination, the authors surveyed the final scaffolds and contigs using BlobTools. The result "All scaffolds and contigs had a top hit match to C. gigas" does mean that major parts of each scaffold/contig are derived from the oyster genome DNA. The result, however, does not confirm that 100% of sequences are from the oyster because this method cannot detect pieces of contaminant sequences in a final scaffolds/contigs. In other words, contaminant sequences may be overlooked if chimeric scaffolds/contigs of the oyster and contaminant genomes were faultily generated by the assembly process.
To solve this issue, I would suggest to analyze raw PacBio reads using BlobTools. If contaminant sequences are detected, the authors may want to map these contaminant reads to the final assembly to see if they were incorporated in the assembly or not.
Response: We thank the reviewer for the comment and agree that Blobtools was not a suitable approach to detect contaminants in our genome assembly. However, and although we appreciate the suggestion of using Blobtools to analyse the raw PacBio reads, we think that it would neither yield good results due to the high intrinsic error rates of these reads. Instead, to detect contaminant sequences in the oyster genome assembly we opted for using Conterminator (https://github.com/martin-steinegger/conterminator), a software that detects cross-kingdom contamination based on an all-against-all sequence comparison, including at an individual contig level. No significant evidence of contaminants were detected in the Pacific oyster genome assembly. However, we accept the fact that our analysis may be limited by the underrepresentation of potential contaminants of aquatic origin in the NCBI nucleotide database.
The sentence describing the evaluation of the assembly using Blobtools was removed from the manuscript (the associated supplementary figure was also removed) and the following sentence was added to the "Methods" section, "e) Quality assessment of reference genome" sub-section in

replacement:
“Firstly, the C. gigas genome assembly was screened for contaminant DNA from a different taxa using Conterminator (Steinegger and Salzberg 2020). The search was done against the nt NCBI database (downloaded Dec 2020) by ignoring unclassified sequences (taxonomy ID 12908), other sequences (28384) and artificial sequences (81077). No evidence of contamination with foreign DNA from a different taxon was detected in the assembly.”

3)Repeat elements vs genes (P.14)
It is not clear how correlations between "the total number of repeat elements" (in each scaffold?) and "gene density" (number of genes per specific sequence window size?) were estimated. I would suggest to describe the method specifically.
Response: The following paragraph has been added to the “Methods” section, “g) Repeat element annotation” sub-section of the manuscript for clarity:
“In general, an inverse relationship between the total number of repeat elements and gene density was observed across 100-kb (non-overlapping) genomic windows in the chromosome-level scaffolds (Figure 3 d-e). If a genomic feature overlapped two windows, the feature was counted towards the interval with the highest length coverage.”

Reviewer #2: This chromosome-scale draft genome of the Pacific oyster (Crassostrea gigas) offers a useful resource for studies of this globally important species. This manuscript is overall nicely written and well presented. The assembly statistics offer a substantial improvement from the original C. gigas draft along with an increase in the number of annotated genes in-line with more recent studies. The assignment of chromosome number to karyotype will also be useful for future comparative studies. The data supporting this manuscript is presently available and although another highly-contiguous genome for this species has been already constructed, it will nonetheless add value to further comparative genomics studies of bivalves.

I have 2 suggestions for additional information that I feel would improve the manuscript.
1) It would be interesting for future users of these data to know more details about the chosen organism. Was the farmed single female oyster from a specifically bred line (disease resistance / growth etc.) or not? There is mention of outbreeding, I feel some more detail of this strategy would be useful.
Response: Additional information explaining the origin of the individual used for sequencing has been added to the “Methods” section, “a) Sample collection and sequencing” sub-section of the manuscript:
“Guernsey Sea Farms is one of the primary suppliers of spat to the UK industry, and has maintained lines of oysters since the early 2000s, when oysters were initially import from British Columbia (Canada), via Seasalter (Whitstable, UK). The stock was later supplemented with genetic material from the Conwy Fisheries Laboratory (UK), which was originally sourced from Japan (Miyagi, Hiroshima and Kumamoto) and the United States (Oregon). These stocks have all been interbred with no specific maintenance of lines.”

2) There appears to be no mention of the mitochondrial genome sequence and it is not specified in the genbank submission. With the long PacBio read lengths, this sequence should have almost been read out in full and the short-read alignment coverage data should have made it easy to identify. A reference should be made to the mitogenome sequence in the assembly.
Response: Indeed, we did sequence the complete mitochondrial genome. However, we were not able to add it to the current version of the reference genome assembly (RefSeq GCF_902806645.1) afterwards. To do so, we would have needed to upload another version of the assembly, which would require re-annotation. Instead, the mitochondrial genome was upload separately and made available online in the Mendeley Data repository (http://dx.doi.org/10.17632/khnhxk38jt.1).

The following sentence was added to the “Methods” section, “d) Chromosome-level assembly using Hi-C and linkage map data” sub-section:
“In addition, the complete mitochondrial genome of C. gigas was assembled and is available online in the Mendeley Data repository [56].”

The following sentence was added to the “Availability of supporting data” section:
“The complete mitochondrial genome is hosted in Mendeley Data, http://dx.doi.org/10.17632/khnhxk38jt.1.”

Minor comments include:
Sentence "633 Mb, with a scaffold and contig N50 of 57 and 0.7 Mb and, respectively." The last "and"

should be deleted.
Response: The typo was corrected.

Table 1; GO and KO abbreviations should be defined for readability.
Response: The following sentence has been added to the header of Table 1 for clarity.
“GO: Gene Ontology annotation; KO: KEGG Orthology annotation.”

References

Pflug, J. M., V. R. Holmes, C. Burrus, J. S. Johnston and D. R. Maddison (2020). "Measuring Genome Sizes Using Read-Depth, k-mers, and Flow Cytometry: Methodological Comparisons in Beetles (Coleoptera)." G3: Genes|Genomes|Genetics 10(9): 3047-3060.

Steinegger, M. and S. L. Salzberg (2020). "Terminating contamination: large-scale search identifies more than 2,000,000 contaminated entries in GenBank." Genome Biology 21(1): 115.

Close
