# Supplementary material for: A chromosome-level genome assembly for the Pacific oyster Crassostrea gigas
Source: Gigascience. 2021 Mar 25;10(3):giab020. doi: 10.1093/gigascience/giab020 (PMC7992393; doi:10.1093/gigascience/giab020)
Supplement: giab020_Reviewer_1_Report_Original_Submission — Takeshi Takeuchi, Ph.D -- 11/9/2020 Reviewed [file giab020_reviewer_1_report_original_submission.pdf]

Reviewer Report

Title: A chromosome-level genome assembly for the Pacific oyster (Crassostrea gigas)

Version: Original Submission **Date:** 11/9/2020

Reviewer name: Takeshi Takeuchi, Ph.D

Reviewer Comments to Author:

PeÃ±aloza et al. sequenced the genome of the Pacific oyster Crasostrea gigas using PacBio long-read sequencing technology and established a chromosome-level assembly by applying Hi-C scaffolding and a high density SNP linkage map. The genome assembly is significantly contiguous compare to previously-published draft assembly of the same species. This new reference genome is a desirable resource, therefore worth to be published in the journal GigaScience after resolving some concerns below.
1)Genome size (P.7)
I don't think it is reasonable to take the midpoint between the results of the k-mer analysis and the flow cytometry measurement. K-mer analysis can considerably underestimate genome size if the genome is rich in repeat sequences, because repeats increase mean coverage of k-mers. Thus, the genome size of 640Mb, measured experimentally, should be more reliable.
2)Assessment of DNA contamination (P.11)
As the genomic DNA was extracted from the gill tissue, where considerable amount of microorganisms may be trapped by the filter-feeding bivalve, contaminant DNA sequences should be carefully removed from the raw reads or the assembly if present.
In order to check contamination, the authors surveyed the final scaffolds and contigs using BlobTools. The result "All scaffolds and contigs had a top hit match to C. gigas" does mean that major parts of each scaffold/contig are derived from the oyster genome DNA. The result, however, does not confirm that 100% of sequences are from the oyster because this method cannot detect pieces of contaminant sequences in a final scaffolds/contigs. In other words, contaminant sequences may be overlooked if chimeric scaffolds/contigs of the oyster and contaminant genomes were faultily generated by the assembly process.
To solve this issue, I would suggest to analyze raw PacBio reads using BlobTools. If contaminant sequences are detected, the authors may want to map these contaminant reads to the final assembly to see if they were incorporated in the assembly or not.
3)Repeat elements vs genes (P.14)
It is not clear how correlations between "the total number of repeat elements" (in each scaffold?) and "gene density" (number of genes per specific sequence window size?) were estimated. I would suggest to describe the method specifically.

Level of Interest

Please indicate how interesting you found the manuscript: Choose an item.

Quality of Written English

Please indicate the quality of language in the manuscript: Choose an item.

Declaration of Competing Interests

Please complete a declaration of competing interests, considering the following questions:

- Have you in the past five years received reimbursements, fees, funding, or salary from an organisation that may in any way gain or lose financially from the publication of this manuscript, either now or in the future?
- Do you hold any stocks or shares in an organisation that may in any way gain or lose financially from the publication of this manuscript, either now or in the future?
- Do you hold or are you currently applying for any patents relating to the content of the manuscript?
- Have you received reimbursements, fees, funding, or salary from an organization that holds or has applied for patents relating to the content of the manuscript?
- Do you have any other financial competing interests?
- Do you have any non-financial competing interests in relation to this paper?

If you can answer no to all of the above, write 'I declare that I have no competing interests' below. If your reply is yes to any, please give details below.

I declare that I have no competing interests.

I agree to the open peer review policy of the journal. I understand that my name will be included on my report to the authors and, if the manuscript is accepted for publication, my named report including any attachments I upload will be posted on the website along with the authors' responses. I agree for my report to be made available under an Open Access Creative Commons CC-BY license (http://creativecommons.org/licenses/by/4.0/). I understand that any comments which I do not wish to be included in my named report can be included as confidential comments to the editors, which will not be published.

Choose an item.

To further support our reviewers, we have joined with Publons, where you can gain additional credit to further highlight your hard work (see: https://publons.com/journal/530/gigascience). On publication of this paper, your review will be automatically added to Publons, you can then choose whether or not to claim your Publons credit. I understand this statement.

Yes Choose an item.
