# Supplementary material for: A chromosome-level genome assembly for the Pacific oyster Crassostrea gigas
Source: Gigascience. 2021 Mar 25;10(3):giab020. doi: 10.1093/gigascience/giab020 (PMC7992393; doi:10.1093/gigascience/giab020)
Supplement: giab020_Reviewer_2_Report_Original_Submission — Daniel Powell -- 11/15/2020 Reviewed [file giab020_reviewer_2_report_original_submission.pdf]

Reviewer Report

Title: A chromosome-level genome assembly for the Pacific oyster (Crassostrea gigas)

Version: Original Submission **Date:** 11/15/2020

Reviewer name: Daniel Powell

Reviewer Comments to Author:

This chromosome-scale draft genome of the Pacific oyster (Crassostrea gigas) offers a useful resource for studies of this globally important species. This manuscript is overall nicely written and well presented. The assembly statistics offer a substantial improvement from the original C. gigas draft along with an increase in the number of annotated genes in-line with more recent studies. The assignment of chromosome number to karyotype will also be useful for future comparative studies. The data supporting this manuscript is presently available and although another highly-contiguous genome for this species has been already constructed, it will nonetheless add value to further comparative genomics studies of bivalves.

I have 2 suggestions for additional information that I feel would improve the manuscript.

1) It would be interesting for future users of these data to know more details about the chosen organism. Was the farmed single female oyster from a specifically bred line (disease resistance / growth etc.) or not? There is mention of outbreeding, I feel some more detail of this strategy would be useful.

2) There appears to be no mention of the mitochondrial genome sequence and it is not specified in the genbank submission. With the long PacBio read lengths, this sequence should have almost been read out in full and the short-read alignment coverage data should have made it easy to identify. A reference should be made to the mitogenome sequence in the assembly.

Minor comments include:

Sentence "633 Mb, with a scaffold and contig N50 of 57 and 0.7 Mb and, respectively." The last "and" should be deleted.

Table 1; GO and KO abbreviations should be defined for readability.

Level of Interest

Please indicate how interesting you found the manuscript: Choose an item.

Quality of Written English

Please indicate the quality of language in the manuscript: Choose an item.

Declaration of Competing Interests

Please complete a declaration of competing interests, considering the following questions:

- Have you in the past five years received reimbursements, fees, funding, or salary from an organisation that may in any way gain or lose financially from the publication of this manuscript, either now or in the future?
- Do you hold any stocks or shares in an organisation that may in any way gain or lose financially from the publication of this manuscript, either now or in the future?
- Do you hold or are you currently applying for any patents relating to the content of the manuscript?
- Have you received reimbursements, fees, funding, or salary from an organization that holds or has applied for patents relating to the content of the manuscript?
- Do you have any other financial competing interests?
- Do you have any non-financial competing interests in relation to this paper?

If you can answer no to all of the above, write 'I declare that I have no competing interests' below. If your reply is yes to any, please give details below.

I declare that I have no competing interests.

I agree to the open peer review policy of the journal. I understand that my name will be included on my report to the authors and, if the manuscript is accepted for publication, my named report including any attachments I upload will be posted on the website along with the authors' responses. I agree for my report to be made available under an Open Access Creative Commons CC-BY license (http://creativecommons.org/licenses/by/4.0/). I understand that any comments which I do not wish to be included in my named report can be included as confidential comments to the editors, which will not be published.

Choose an item.

To further support our reviewers, we have joined with Publons, where you can gain additional credit to further highlight your hard work (see: https://publons.com/journal/530/gigascience). On publication of this paper, your review will be automatically added to Publons, you can then choose whether or not to claim your Publons credit. I understand this statement.

Yes Choose an item.
